# Factors influencing survival and mortality among adult Aboriginal Australians with bronchiectasis—A 10-year retrospective study

**DOI:** 10.3389/fmed.2024.1366037

**Published:** 2024-05-07

**Authors:** Subash S. Heraganahally, Claire Gibbs, Shiidheshwar J. Ravichandran, Davaadorj Erdenebayar, Asanga Abeyaratne, Timothy Howarth

**Affiliations:** ^1^Department of Respiratory and Sleep Medicine, Royal Darwin Hospital, Darwin, NT, Australia; ^2^College of Medicine and Public Health, Flinders University, Darwin, NT, Australia; ^3^Darwin Respiratory and Sleep Health, Darwin Private Hospital, Darwin, NT, Australia; ^4^Department of Medicine, Royal Darwin Hospital, Darwin, NT, Australia; ^5^Menzies School of Health Research, Darwin, NT, Australia; ^6^Diagnostic Imaging Center, Kuopio University Hospital, Kuopio, Finland; ^7^Department of Technical Physics, University of Eastern Finland, Kuopio, Finland

**Keywords:** First Nations, chest CT, morbidity, pulmonary, respiratory, Sputum culture, spirometry, severity

## Abstract

**Background:**

The prevalence of bronchiectasis among adult Aboriginal Australians is higher than that of non-Aboriginal Australians. However, despite evidence to suggest higher prevalence of bronchiectasis among Aboriginal people in Australia, there is sparce evidence in the literature assessing clinical parameters that may predict survival or mortality in this population.

**Methods:**

Aboriginal Australians residing in the Top End Health Service region of the Northern Territory of Australia aged >18 years with chest computed tomography (CT) confirmed bronchiectasis between 2011 and 2020 were included. Demographics, body mass index (BMI), medical co-morbidities, lung function data, sputum microbiology, chest CT scan results, hospital admissions restricted to respiratory conditions and all-cause mortality were assessed.

**Results:**

A total of 459 patients were included, of whom 146 were recorded deceased (median age at death 59 years). Among the deceased cohort, patients were older (median age 52 vs. 45 years, *p* = 0.023), had a higher prevalence of chronic obstructive pulmonary disease (91 vs. 79%, *p* = 0.126), lower lung function parameters (median percentage predicted forced expiratory volume in 1 s 29 vs. 40%, *p* = 0.149), a significantly greater proportion cultured non-*Aspergillus* fungi (65 vs. 46%, *p* = 0.007) and *pseudomonas* (46 vs. 28%, *p* = 0.007) on sputum microbiology and demonstrated bilateral involvement on radiology. In multivariate models advancing age, prior *pseudomonas* culture and Intensive care unit (ICU) visits were associated with increased odds of mortality. Higher BMI, better lung function on spirometry, prior positive sputum microbiology for *Haemophilus* and use of inhaled long-acting beta antagonist/muscarinic agents may have a favourable effect.

**Conclusion:**

The results of this study may be of use to stratify high risk adult Aboriginal patients with bronchiectasis and to develop strategies to prevent future mortality.

## 1 Introduction

Bronchiectasis is a chronic pulmonary condition that is clinically characterised by a vicious cycle of recurrent lower respiratory tract infections and airway inflammation (1, 2). Globally, there is emerging evidence to suggest that presence of bronchiectasis is associated with overall higher mortality rates (3–9). In the Australian context, approximately 3.3% of the population self-identify as of Aboriginal and/or Torres Strait Islander descent (from here on “Indigenous” is used to refer to global First nations populations, while “Aboriginal Australian/ population/patients/people” is used to specifically refer to Australians First Nations population) and the Northern Territory (NT) of Australia has the highest proportion of Australian Aboriginal people in comparison to all other Australian states and territories (10). Chronic respiratory disorders are reported to be highly prevalent among the adult Aboriginal Australian population (11, 12), and more so among those residing in the NT of Australia. In particular, prevalence of bronchiectasis is noted to be substantially higher among Aboriginal Australians compared to non-Aboriginal Australians (13, 14). Furthermore, hospital admission rates and overall mortality secondary to chronic respiratory disorders, including for bronchiectasis, are significantly higher among adult Aboriginal Australians in comparison to their non-Aboriginal counterparts (15–20).

In other diverse non-Indigenous ethnic populations, studies have demonstrated several factors which influence survival and mortality amongst patients with bronchiectasis such as, but not limited to lung function parameters, sputum microbiology, body mass index (BMI) and therapeutic interventions such as respiratory airway clearance (21–23). However, despite the high prevalence of bronchiectasis among Aboriginal Australians, there is scant evidence in the literature determining if these same factors are associated with survival or mortality in this population. The high prevalence of comorbidities, reduced lung function parameters (11–20, 24, 25), and unique environmental context of Aboriginal Australians which may predispose them to colonisation by other micro-organisms may indicate that other factors should be considered when predicting mortality risk. Hence, it is reasonable to explore those potential clinical parameters that may be influential for survival or mortality among adult Aboriginal Australians with bronchiectasis. This may lead on to identifying relevant clinical data for future interventions which would aid in reducing on-going adverse health consequences amongst adult Aboriginal patients suffering from bronchiectasis. Therefore, the aim of this study is to investigate and identify relevant clinical parameters that may indicate or influence survival and mortality in an adult Aboriginal Australian cohort diagnosed to have bronchiectasis over a 10-year study period (2011–2020) in the Top End Health Service (TEHS) region of the NT of Australia.

## 2 Materials and methods

### 2.1 Setting and study participants

This study was conducted at the respiratory and sleep division based at the Royal Darwin Hospital, a university affiliated tertiary care teaching hospital and Darwin Respiratory and Sleep Health, Darwin Private Hospital, within the TEHS, NT region of Australia. This study is a part of a larger project examining various aspects of bronchiectasis disease profiles among the adult Aboriginal population residing in the TEHS health district of the NT of Australia, which is inclusive of all adult Australian Aboriginal patients aged ≥18 years identified to have bronchiectasis via chest Computed tomography (CT) scan between 2011 and 2020.

### 2.2 Ethics

This study was approved by the Human Research Ethics governance/committee of the TEHS, NT and Menzies School of Health Research (Reference: HREC; 2019-3547). The authors acknowledge the rights of Australian Aboriginal people involved in this study, and as such conducted and reported according to strengthening and reporting of health research involving Aboriginal people (26), including consultations with institute Aboriginal representatives.

### 2.3 Clinical data assessed

Baseline demographics, age, sex, including smoking status and BMI when available were recorded. Patients usual place of residence as per community/suburb/postcode were collected, with further categorisation into the four health districts of the Top End (Darwin Urban, Darwin Rural, East Arnhem and Katherine), and by community when communities had >10 bronchiectasis cases were present (27). Presence of respiratory conditions alongside bronchiectasis and other medical comorbidities, including inhaled pharmacotherapy use [short-acting/long-acting beta antagonist (SABA/LABA), short-acting/long-acting muscarinic agents (SAMA/LAMA), inhaled corticosteroids (ICS)] were recorded. Details of chest CT scan findings, lung function test results [spirometry, forced vital capacity (FVC) and forced expiratory volume in 1 s (FEV_1_)] and sputum microbiology results were also collected. Only respiratory related hospital admissions were extracted for assessment. All clinical parameters for this study were assessed via individual patients’ electronic medical records. Mortality data was extracted through the hospital information system, death registry linkage was not utilised, and all-cause mortality was documented up until 31st December 2020. Further details on methods and study design are available form a recent report from our centre (27). The primary outcome in this study was all-cause mortality.

### 2.4 Statistical analysis

Data were presented as median [interquartile range (IQR)] for continuous parameters, or number (%) for categorical parameters. Differences in demographic and clinical characteristics, radiological extent, hospitalisations and sputum cultures between surviving and deceased patients were tested via univariate logistic regression (categorical parameters) or quantile regression (continuous parameters). Stepwise Cox and quantile multivariate regressions were performed to identify factors associated with mortality and with age of death, respectively. The models included demographics (age, sex and residence location using Darwin area as reference), presence of comorbidities (restricted to those experienced by >10 patients in this cohort), radiological extent (bilateral involvement and ≥3 lobes effected), hospitalisation history (any hospitalisations, total time in hospital, any intensive care unit (ICU) visits), sputum results (presence of *haemophilus*, *streptococcus*, *moraxella*, *staphylococcus*, *aspergillus*, non-*Aspergillus* fungi, *klebsiella*, *mycobacterium* or *burkholderia*), and pharmacotherapy. A second multivariate Cox regression model was created (see Supplementary material 2) for mortality including BMI, current smoking and lung function, as only one-third of patients in our cohort had these data available. In the stepwise models, parameters with a *p*-value < 0.1 were considered for further analyses. Multicollinearity of included parameters was checked via Pearsons *R*^2^, and pairs with an *R*^2^ > 0.5 sequentially excluded until the models with the best fit were found. Results were presented as hazard ratios (HRs) (95% confidence intervals (CIs)) or beta (95% CI). All *p*-values were adjusted for multiple hypotheses testing via Romano-Wolf adjustment, utilising 750 bootstrap replications. Analyses were conducted in STATA IC 15 (College Station, Texas).

## 3 Results

### 3.1 Demographic and clinical details

**FIGURE 1:**
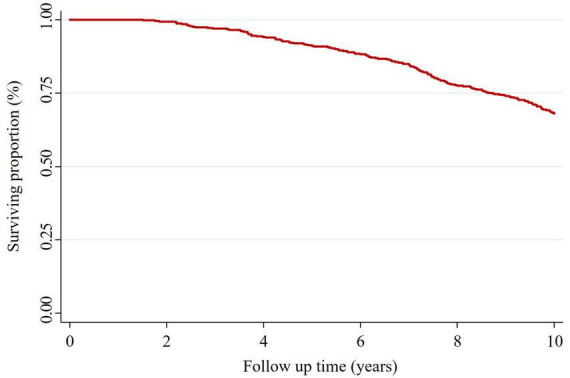
Kaplan-Meier survival graph across the study period (Jan 2011-December 2020).

In total 459 patients were retrospectively enrolled, of which 146 (31.8%) were recorded deceased by 31st of December 2020 (Figure 1). The median follow-up time for the surviving group was 10 years (IQR 10, 10), and for the deceased group was 7.1 years (IQR 4.6, 8.5). Patients who survived during this study period were younger upon enrolment [median 45 years (IQR 38.2, 53.5) vs. 52.2 years (IQR 43.4, 59.5), *p* = 0.023], had higher FEV_1_ [40% (IQR 31, 35) vs. 29% predicted (IQR 24, 40)] and a lower prevalence of chronic obstructive pulmonary disease (COPD) (79 vs. 91%), lung cancer (3 vs. 7%), hypertension (58 vs. 73%), chronic kidney disease (CKD) (36 vs. 50%) and heart failure (7 vs. 12%) than patients who were recorded deceased (Table 1). However, following Romano-Wolf adjustment, aside from age, there were no statistically significant differences in relation to clinical, comorbidities nor inhaled pharmacotherapy prescriptions between the two groups. There were also no significant differences noted in mortality between health districts, nor at the community level (Supplementary material 1).

**TABLE 1:** Demographic and clinical characteristics of surviving and deceased patients.

Clinical parameters	Surviving (*n* = 313)	Deceased (*n* = 146)	RW-p
Age enrolment	45.02 (38.23, 53.51)	52.17 (43.41, 59.5)	0.007*
Age at death	–	59.17 (49.73, 67.42)	–
Sex (female)	173 (55.3%)	81 (55.5%)	0.987
BMI (kg/m^2^)^	23.45 (19.57, 27.7)	21.67 (18.15, 25.12)	0.848
Darwin Urban	19 (6.1%)	15 (10.3%)	0.841
Darwin Rural	156 (49.8%)	70 (48.0%)	0.987
East Arnhem	78 (24.9%)	24 (16.4%)	0.649
Katherine	60 (19.2%)	37 (25.3%)	0.848
Never smoker^	15 (13.3%)	7 (18.9%)	0.960
Former smoker^	53 (46.9%)	11 (29.7%)	0.775
Current smoker^	45 (39.8%)	19 (51.4%)	0.861
FVC (% predicted)^	53 (42, 65)	44.5 (34, 53.5)	0.841
FEV_1_ (% predicted)^	40 (31, 55)	29 (24, 40)	0.093
FEV_1_/FVC^	0.66 (0.51, 0.76)	0.57 (0.43, 0.69)	0.828
COPD	247 (78.9%)	133 (91.1%)	0.086
Asthma	76 (24.3%)	41 (28.1%)	0.960
Lung cancer	8 (2.6%)	10 (6.8%)	0.576
HTN	182 (58.1%)	107 (73.3%)	0.093
T2DM	148 (47.3%)	76 (52.1%)	0.960
CKD	111 (35.5%)	73 (50%)	0.106
CAD	102 (32.6%)	58 (39.7%)	0.848
HF	21 (6.7%)	18 (12.3%)	0.676
SABA	191 (61%)	94 (64.4%)	0.987
SAMA	18 (5.8%)	15 (10.3%)	0.828
LABA	188 (60.1%)	91 (62.3%)	0.987
LAMA	147 (47%)	64 (43.8%)	0.987
ICS	170 (54.3%)	82 (56.2%)	0.987

*Indicates *p*-value < 0.05.

^BMI and lung function data was available for 169 patients (*n* = 129 and 40 surviving and deceased, respectively), and smoking data available for 150 patients (*n* = 113 and 37 surviving and deceased, respectively). BMI, body mass index; FVC, forced vital capacity; FEV_1_, forced expiratory volume in 1 s; COPD, chronic obstructive pulmonary disease; HTN, hypertension; T2DM, type 2 diabetes mellitus; CKD, chronic kidney disease; CAD, coronary artery disease; HF, heart failure; SABA, short acting beta antagonist; SAMA, short acting muscarinic agent; LABA, long acting beta antagonist; LAMA, long acting muscarinic agent; ICS, Inhaled corticosteroids; RW-p, Romano-Wolf adjusted *p*-value.

### 3.2 Chest CT findings and mortality

Chest CT scans demonstrated that the left lower lobe was the most commonly affected in both surviving (74%) and deceased (73%) cohorts, followed by the right lower lobe (59 and 69%, respectively) (Table 2). The left upper lobe appeared less commonly affected in the surviving cohort (19%) compared to the deceased cohort (27%), as was bilateral involvement (72 and 78%, respectively), however, the differences did not reach statistical significance.

**TABLE 2:** Radiological extent of bronchiectasis between surviving and deceased patients.

Location of bronchiectasis on chest CT scan	Surviving (*n* = 313)	Deceased (*n* = 146)	RW-p
RLL	185 (59.1%)	100 (68.5%)	0.305
RML	136 (43.5%)	60 (41.1%)	0.934
RUL	79 (25.2%)	47 (32.2%)	0.464
LLL	231 (73.8%)	106 (72.6%)	0.954
Lingula	89 (28.4%)	41 (28.1%)	0.954
LUL	60 (19.2%)	40 (27.4%)	0.305
Bilateral	225 (71.9%)	114 (78.1%)	0.490
Median number of lobes affected	2 (1, 3)	2 (2, 3)	1.000
≥3 lobes effected	110 (35.1%)	58 (39.7%)	0.755

CT, computed tomography; RLL, right lower lobe; RML, right middle lobe; RUL, right upper lobe; LLL, left lower lobe; LUL, left upper lobe; RW-p, Romano-Wolff adjusted *p*-value.

### 3.3 Sputum cultures and mortality data

Sputum cultures were available for 425 patients [283 surviving (90.4%), 142 deceased (97.3%)]. *Haemophilus* was the most commonly cultured sputum overall, followed by non-*Aspergillus* fungi. Among those surviving, *haemophilus* (65 vs. 58%, *p* = 0.593) was the most commonly cultured organism, while among those deceased a greater proportion recorded non-*Aspergillus* fungi (65 vs. 46%, *p* = 0.007). *Pseudomonas* was also cultured less often in the surviving cohort compared to the deceased (28 vs. 46%, *p* = 0.007) (Table 3).

**TABLE 3:** Sputum cultures for surviving and deceased patients.

Sputum microbiology	Surviving (*n* = 283)	Deceased (*n* = 142)	RW-p
Haemophilus	183 (64.7%)	82 (57.7%)	0.593
non-*Aspergillus fungi*	131 (46.3%)	92 (64.8%)	0.007*
Streptococcus	96 (33.9%)	54 (38%)	0.893
Pseudomonas	78 (27.6%)	65 (45.8%)	0.007*
Staphylococcus	36 (12.7%)	28 (19.7%)	0.286
Mycobacterium	35 (12.4%)	18 (12.7%)	0.971
Aspergillus	21 (7.4%)	16 (11.3%)	0.600
Klebsiella	18 (6.4%)	11 (7.7%)	0.936
Burkholderia	13 (4.6%)	7 (4.9%)	0.971
Other	280 (98.9%)	138 (97.2%)	0.600

*Indicates *p*-value < 0.05. RW-p, Romano-Wolff adjusted *p*-value. A total of 34 patients did not have sputum data available and were excluded from this table.

### 3.4 Hospitalisations and mortality data

The majority of patients recorded at least one respiratory condition related hospitalisation during the study period, though a lesser proportion of those surviving did so (83%) compared to those deceased (95%) (Table 4). Patients who survived spent significantly less total time in hospital during the study window, with a median 12 days (IQR 3, 34) compared to those deceased with a median 34 days (IQR 12, 69) (*p* = 0.027). Half as many surviving patients recorded time in the ICU (22 vs. 42%, *p* = 0.066) or time on mechanical ventilation (6 vs. 12%, *p* = 0.431).

**TABLE 4:** Hospitalisation information of surviving and deceased patients.

Hospitalisation data	Surviving (*n* = 313)	Deceased (*n* = 146)	RW-p
Any hospitalisations	260 (83.1%)	138 (94.5%)	0.119
Median number of hospitalisations	3 (1, 8)	5 (2, 12)	0.431
Total time in hospital (days)	12 (3, 34)	34 (12, 69)	0.027*
Any ICU	58 (22.3%)	58 (42%)	0.066
Total time in ICU (hours)	112.5 (64, 204)	143.5 (73, 302)	0.735
Any mechanical ventilation	16 (6.2%)	16 (11.6%)	0.431
Total time on ventilation (hours)	48.5 (30, 125)	58 (30.5, 243.5)	0.808

*Indicates *p*-value < 0.05. ICU, intensive care unit; RW-p, Romano-Wolff adjusted *p*-value.

### 3.5 Regression analysis

In the Cox regression model (425 patients included), older age, presence of *Pseudomonas* and recorded ICU visits were associated with increased odds of mortality while cultured *Haemophilus* was associated with reduced odds of mortality (Table 5). In multivariate analysis use of LABA and LAMA were associated with reduced HR for morality, but this was attenuated with multiple hypothesis adjustment. In quantile regression (142 patients included), older age at the start of the study period and LABA prescription were associated with significantly older age at death, though the statistical significance associated with LABA prescription was attenuated following Romano-Wolf adjustment. Cox regression models for patients with lung function data, BMI and smoking data are displayed in Supplementary material 2. In the univariate cox regression model increasing FEV_1_ [HR 0.96 (95% CI 0.94, 0.99), *p* = 0.024] and BMI were associated with reduced odds of mortality [HR 0.92 (95% CI 0.87, 0.98), *p* = 0.051], however, in the multivariate model (*n* = 142) only BMI showed a statistically significant association with mortality [HR 0.88 (95% CI 0.82, 0.954), *p* = 0.017].

**TABLE 5:** Univariate and multivariate Cox regression models for mortality reporting HRs (95% CI) and quantile regression models for age of death reporting betas (95% CI) for parameters included from stepwise regression.

Univariate results	Cox regression for mortality	RW-p	Quantile regression for age of death	RW-p
Age	1.04 (1.03, 1.06)	0.001*	1.00 (0.95, 1.04)	0.001*
COPD	2.3 (1.3, 4.07)	0.016*	–	–
*Haemophilus*	0.8 (0.58, 1.12)	0.479	–	–
non-*Aspergillus fungi*	1.84 (1.31, 2.6)	0.001*	–	–
*Pseudomonas*	1.94 (1.4, 2.71)	0.001*	−0.49 (−6.89, 5.91)	0.912
*Staphylococcus*	1.56 (1.03, 2.36)	0.143	–	–
ICU	2.28 (1.64, 3.18)	0.001*	–	–
LABA	1.04 (0.75, 1.46)	0.802	2.91 (−3.35, 9.16)	0.581
LAMA	0.89 (0.64, 1.23)	0.688	–	–
**Multivariate results**	**Cox regression for** **mortality—(*n* = 425)**	**RW-p**	**Quantile regression for age** **of death—(*n* = 142) Pseudo** ***R*^2^ 0.824**	**RW-p**
Age	1.04 (1.02, 1.06)	0.001*	1.00 (0.96, 1.05)	0.001*
COPD	2.17 (1.13, 4.16)	0.107	–	–
*Haemophilus*	0.6 (0.41, 0.86)	0.037*	–	–
non-*Aspergillus fungi*	1.4 (0.96, 2.03)	−0.128	–	–
*Pseudomonas*	1.71 (1.2, 2.44)	0.027*	−1.00 (−2.05, 0.05)	0.202
*Staphylococcus*	1.64 (1.07, 2.53)	0.107	–	–
ICU	2.28 (1.58, 3.31)	0.001*	–	–
LABA	0.68 (0.47, 1)	0.128	1.72 (0.64, 2.8)	0.108
LAMA	0.67 (0.47, 0.96)	0.107	–	–

The Cox regression model included sputum cultures which were not available for 34 patients, therefore 425/459 patients were included for univariate modelling of these parameters, and in the multivariate model.

*Indicates Romano-Wolf adjusted *p*-value < 0.05. COPD, Chronic obstructive pulmonary disease; ICU, intensive care unit; LABA, long acting beta antagonist; LAMA, long acting muscarinic agent; RW-p, Romano-Wolff adjusted *p*-value.

## 4 Discussion

To the best of the authors knowledge, this is the first study to demonstrate potential clinical parameters that may influence survival or future mortality amongst an adult Aboriginal Australian population, particularly among those residing in the Top End, NT of Australia. This study has illustrated that increasing age, prior need for ICU admissions, and prior positive sputum microbiology demonstrating *Pseudomonas may* increase the risk of mortality. On the other hand, a higher BMI, better lung function on spirometry and prior positive sputum microbiology demonstrating *Haemophilus* may be associated with a favourable outcome.

Across various ethnically diverse non-Indigenous populations, hospitalisation and mortality data have been well documented in the literature (3–9, 21–23, 28–31). Although the available reports suggest that the burden of chronic respiratory disorders is much higher among global First Nations Indigenous people (including higher heath care utilisation and hospital admission rates), there is scant evidence pertaining to factors that may predict mortality or survival among First Nations Indigenous people (20, 32–38).

In comparison to international bronchiectasis cohorts, the current study cohort showed some unique clinical features. Most notably, the median age of death was 59.2 years, whereas in international data most deaths are among those aged >70 years (3). However, similar to as has been reported previously (3, 22, 39), we also noted a lower BMI in the deceased group at a median 22 kg/m^2^. The reason for a lower BMI being associated with mortality is unclear. It is reasonable to speculate that the resting energy expenditure and higher basal metabolic rate due to chronic respiratory tract infection, alongside chronic airway inflammation may be contributing to a lower BMI among patients with bronchiectasis as observed among patients with COPD (40, 41).

Among patients with bronchiectasis, multimorbidity, and especially respiratory comorbidities have been associated with significantly greater morbidity and mortality (4–6). Coexisting COPD in particular is shown to have a much worse prognosis (42). Among our cohort there was a significant prevalence of COPD, particularly among the deceased cohort at 91%, however, 79% of surviving patients were also noted to have COPD. This significant presence of disease amongst both surviving and deceased cohorts limits its ability to be used as a prognostic marker as it is in other populations.

In relation to laboratory parameters, lower lung function values, presence of *pseudomonas* on sputum microbiology and CT evidence of multi-lobar involvement are shown to be associated with poorer long-term prognosis among patients with bronchiectasis (3–9, 21). Among the deceased cohort in our study, lung function values were reduced (medians for FVC 45 vs. 53%, FEV_1_ 29 vs. 40%, FEV_1_/FVC ratio 57 vs. 66 deceased vs. surviving, respectively) though this did not reach statistical significance. Although, this may not be statistically significant, it may have clinical relevance. Similar to what is observed in previous reports (6, 43), *Pseudomonas* was cultured more commonly among deceased patients than those surviving (45.8 vs. 27.6%). However, in our study in addition to *pseudomonas*, presence of non-*Aspergillus* fungi on sputum microbiology was significantly more common among deceased patients (64.8 vs. 46.3%). Further prospective studies may be useful to explore microbiology data in this population. The effects of therapeutic interventions, such as inhaled antibiotics (44) on survival and mortality has not been widely explored in the adult Aboriginal Australian patients with bronchiectasis. Further studies may be useful to investigate the beneficial effects of therapeutic modalities such as inhaled antibiotics, including N- Acetylcysteine in Indigenous patients (45). Use of ICS among patients with bronchiectasis is controversial (46, 47). Nonetheless, in our study more than half of the patients recorded an ICS prescription. ICS did not show any positive or negative association with mortality in our study. LABA and LAMA prescription however, was associated with reduced odds of mortality, and LABA prescription with an older age at death in multivariate regressions, though statistical significance of both was attenuated on multiple hypotheses adjustment. Nevertheless, in view of previous studies demonstrating positive impact on the use of LAMA among patients with bronchiectasis (48, 49), it is reasonable to assume that use of LAMA may have some favourable impact on survival in our study patients.

Among Aboriginal Australian patients, hospital admission rates secondary to respiratory conditions are reported to be higher than among non-Aboriginal Australian patients (50). In our study we noted that among the deceased cohort there was a significantly longer median time spent in hospital (34 compared to 12 days). Furthermore, a greater proportion among the deceased cohort required ICU care at some point (42 vs. 22%). ICU visits were one of the few parameters to retain a statistically significant association with increased mortality following multiple hypothesis correction, indicating that this factor should be considered as a marker for poor long-term prognosis among Aboriginal patients with bronchiectasis.

This study has demonstrated that several classic clinical parameters used for predicting survival, exacerbations/hospital admissions or mortality amongst adults with bronchiectasis may not be as applicable to Aboriginal Australians as in other non-Indigenous population (51–53). This is due to a significant background presence of comorbidities, high smoking rates, reduced lung function parameters, significantly younger age and generally lower BMI within this cohort. Given the disparity noted in some of the clinical parameters in comparison to other ethnic non-Indigenous patients as noted in this study and in general (54–69), moving forwards, it may be time to invest in establishing Indigenous specific bronchiectasis assessment and severity classification tool. This will be very valuable to identify high risk adult Aboriginal patients with bronchiectasis in order to guide clinical decision making and early interventions to reduce overall morbidity and mortality.

## 5 Limitation

This study’s outcomes pertain to Aboriginal Australian people residing in the TEHS region of the NT of Australia and the results represented in this study cannot be generalised to the wider Aboriginal populations in Australia or for Indigenous people globally. Spirometry data and BMI values were not available for all patients, hence would have introduced a bias in the outcome observed. We also did not have data to represent therapeutic interventions other than inhaled pharmacotherapy use and moreover, we did not have dates of prescription of inhaled pharmacotherapy, nor to assess dosages or adherence to medication which would be significant confounders. Furthermore, with a high presence of COPD, CAD, and CKD in this population, which was observed among both deceased and surviving cohorts, it is uncertain if bronchiectasis is a primary or a secondary cause contributing to mortality. Nonetheless, this is the first study to assess factors predicting survival and mortality in an Aboriginal population, which could be useful to compare for any future similar studies in other Indigenous global populations.

## 6 Conclusion

Among adult Aboriginal Australian patients diagnosed to have bronchiectasis, advancing age, cultured *Pseudomonas* and prior ICU visits are strongly associated with mortality. Higher BMI, better lung function parameters, prior positive sputum microbiology for *Haemophilus* and use of LABA and LAMA may have a favourable effect. However, further studies are warranted in larger cohorts to determine if these findings are replicable. Furthermore, it is clear that efforts must be made to establish bronchiectasis severity assessment tools specific to Indigenous people to stratify high risk patients, so that interventions can be implemented to prevent mortality.

## Data availability statement

The original contributions presented in the study are included in the article/Supplementary material, further inquiries can be directed to the corresponding author.

## Ethics statement

The studies involving humans were approved by the Human Research Ethics Governance/Committee of the TEHS, NT and Menzies School of Health Research (Reference: HREC; 2019-3547). The studies were conducted in accordance with the local legislation and institutional requirements. The Ethics Committee/Institutional Review Board waived the requirement of written informed consent for participation from the participants or the participants’ legal guardians/next of kin because Due to this research being a retrospective study–informed consent was waived by the Ethics Committee.

## Author contributions

SH: Conceptualization, Data curation, Formal analysis, Funding acquisition, Investigation, Methodology, Project administration, Resources, Software, Supervision, Validation, Visualization, Writing – original draft, Writing – review and editing. CG: Conceptualization, Data curation, Investigation, Methodology, Resources, Software, Validation, Visualization, Writing – original draft, Writing – review and editing. SR: Conceptualization, Data curation, Investigation, Resources, Visualization, Writing – review and editing. DE: Conceptualization, Data curation, Investigation, Resources, Validation, Visualization, Writing – review and editing. AA: Conceptualization, Data curation, Investigation, Methodology, Resources, Software, Validation, Visualization, Writing – original draft, Writing – review and editing. TH: Conceptualization, Data curation, Formal analysis, Investigation, Methodology, Project administration, Resources, Software, Supervision, Validation, Visualization, Writing – original draft, Writing – review and editing.
