# Supplementary material for: Factors influencing survival and mortality among adult Aboriginal Australians with bronchiectasis—A 10-year retrospective study
Source: Front Med (Lausanne). 2024 May 7;11:1366037. doi: 10.3389/fmed.2024.1366037 (PMC11106411; doi:10.3389/fmed.2024.1366037)
Supplement: Supplementary file 1 [file Data_Sheet_1.pdf]

Supplement 1. Health district and community locations of surviving and deceased patients

**Health District**	**Surviving (n=313)**	**Deceased (n=146)**	**RW-p**
Darwin Urban	19 (6.1%)	15 (10.3%)	0.199
Darwin Rural	156 (49.8%)	70 (47.9%)	0.550
East Arnhem	78 (24.9%)	24 (16.4%)	0.199
Katherine	60 (19.2%)	37 (25.3%)	0.265
**Community**	**Surviving (n=215)**	**Deceased (n=103)**	
A (n=12)	6 (2.8%)	6 (5.8%)	0.900
B (n=28)	18 (8.4%)	10 (9.7%)	0.988
C (n=16)	10 (4.7%)	6 (5.8%)	0.988
D (n=22)	12 (5.6%)	10 (9.7%)	0.850
E (n=14)	12 (5.6%)	2 (1.9%)	0.875
F (n=41)	23 (10.7%)	18 (17.5%)	0.850
G (n=23)	14 (6.5%)	9 (8.7%)	0.975
H (n=17)	12 (5.6%)	5 (4.9%)	0.988
I (n=82)	58 (27%)	24 (23.3%)	0.988
J (n=12)	9 (4.2%)	3 (2.9%)	0.988
K (n=16)	14 (6.5%)	2 (1.9%)	0.513
L (n=20)	15 (7%)	5 (4.9%)	0.975
M (n=15)	12 (5.6%)	3 (2.9%)	0.988

* indicates p-value <0.05

Communities' names have been assigned a letter to maintain patient confidentiality. Only remote communities with >10 cases were included for this analysis, therefore total numbers are smaller than numbers of the total cohort.

Abbreviations: RW-p, Romano-Wolf adjusted p-value

Supplement 2. Cox univariate and multivariate regression models for mortality reporting HRs (95% CI) including BMI, smoking history and lung function.

Clinical data	Multivariate regression (n=142)	Adjusted p-value	Univariate regressions	Adjusted p-value
Age	1.06 (1.02, 1.09)	0.073	1.04 (1.03, 1.06)	0.001*
COPD	2.62 (0.3, 22.76)	0.876	2.3 (1.3, 4.07)	0.024*
Haemophilus	0.35 (0.15, 0.8)	0.226	0.8 (0.58, 1.12)	0.538
Non-*Aspergillus* fungi	2.8 (1.21, 6.49)	0.244	1.84 (1.31, 2.6)	0.008*
Pseudomonas	2.4 (1.08, 5.31)	0.343	1.94 (1.4, 2.71)	0.001*
Staphylococcus	1.38 (0.57, 3.37)	0.876	1.56 (1.03, 2.36)	0.170
ICU	2.65 (1.25, 5.61)	0.219	2.28 (1.64, 3.18)	0.001*
LABA	1.29 (0.51, 3.3)	0.876	1.04 (0.75, 1.46)	0.802
LAMA	1.61 (0.71, 3.64)	0.822	0.89 (0.64, 1.23)	0.688
BMI	0.88 (0.82, 0.94)	0.017*	0.92 (0.87, 0.98)	0.051
Smoking history	0.65 (0.25, 1.66)	0.876	0.57 (0.25, 1.3)	0.538
FEV_1	0.97 (0.94, 1)	0.463	0.96 (0.94, 0.99)	0.024*

* indicates Romano-Wolf adjusted p-value <0.05

Comorbid lung cancer was excluded from the stepwise regression for these models as less than 10 patients who fit inclusion criteria for these models had lung cancer recorded.

Abbreviations: BMI, Body mass index; COPD, Chronic obstructive pulmonary disease; ICU, Intensive care unit; LABA, Long acting beta antagonist; LAMA, Long acting muscarinic agent; FEV_1, Forced expiratory volume in one second.
